# High-frequency brain activity and muscle artifacts in MEG/EEG: a review and recommendations

**DOI:** 10.3389/fnhum.2013.00138

**Published:** 2013-04-15

**Authors:** Suresh D. Muthukumaraswamy

**Affiliations:** CUBRIC, School of Psychology, Cardiff UniversityCardiff, UK

**Keywords:** high-frequency activity, muscle artifacts, gamma-band activity, magnetoencephalography, electroencephalography

## Abstract

In recent years high-frequency brain activity in the gamma-frequency band (30–80 Hz) and above has become the focus of a growing body of work in MEG/EEG research. Unfortunately, high-frequency neural activity overlaps entirely with the spectral bandwidth of muscle activity (~20–300 Hz). It is becoming appreciated that artifacts of muscle activity may contaminate a number of non-invasive reports of high-frequency activity. In this review, the spectral, spatial, and temporal characteristics of muscle artifacts are compared with those described (so far) for high-frequency neural activity. In addition, several of the techniques that are being developed to help suppress muscle artifacts in MEG/EEG are reviewed. Suggestions are made for the collection, analysis, and presentation of experimental data with the aim of reducing the number of publications in the future that may contain muscle artifacts.

In recent years high-frequency brain activity in the gamma-frequency band (30–80 Hz) and above has become the focus of a growing body of work in MEG/EEG^1^ research. Although this focus is relatively recent, over 60 years ago, the pioneering work of Moruzzi and Magoun (1949) demonstrated that stimulation of the brainstem reticular formation leads to suppression of slow EEG rhythms and the emergence of low-voltage fast waves; they termed this phenomenon an “activated” EEG state. This conceptualization of the frequency characteristics of cortical activation is still present in modern neuroimaging (Kilner et al., 2005; Mukamel et al., 2007; Magri et al., 2012). With a few notable exceptions, such as Freeman's studies of high-frequency wave packets in the olfactory cortex of rabbits (Freeman, 1975) and, later, monkey visual cortex (Freeman and van Dijk, 1987), and Chatrian's observation of 50 Hz oscillations in human calcarine cortex during visual stimulation (Chatrian et al., 1960), higher frequency activity was relatively unstudied until the influential work of Gray and Singer (Gray et al., 1989; Gray and Singer, 1989) suggested that these frequencies play an important role in cortical information processing. Following this suggestion, the low-pass filters—traditionally set in EEG at around 30 Hz (Niedermeyer and Lopes Da Silva, 2005; Fries et al., 2008) to help prevent aliasing and suppress muscle artifacts—have been increasingly elevated in an attempt to characterize high-frequency activity non-invasively. As was predicted (Bressler, 1990), high-frequency activity has now been found across the neocortex and has been shown to be involved in a plethora of functions, including sensory processing, movement control, memory and attention. Unfortunately, high-frequency neural activity overlaps entirely with the spectral bandwidth of muscle activity (~20–300 Hz). It is becoming appreciated that artifacts of muscle activity may contaminate a number of non-invasive reports of high-frequency activity. Moreover, insufficient reporting of scientific data in some publications make it virtually impossible to tell *post-hoc* whether a particular reported effect is high-frequency activity of neural origin. In this review, the spectral, spatial, and temporal characteristics of muscle artifacts are compared with those described (so far) for high-frequency neural activity. In addition, several of the techniques that are being developed to help suppress muscle artifacts in MEG/EEG are reviewed. As will be shown, no single technique can suppress all muscle artifacts, nor does there exist a single data feature that allows easy discrimination of brain and muscle activity. Suggestions are made for the collection, analysis, and presentation of experimental data with the aim of reducing the number of publications in the future that may contain artifacts.

## The characteristics of muscle artifacts in MEG/EEG

A good starting point is a consideration of the basic spectral properties of the muscles that are most likely to interfere with MEG/EEG recordings. It is well-known that the power spectrum of contracting striated muscle, measured with surface electromyography, shows a bandwidth of 20–300 Hz and that most of the power is in the lower end of this frequency range (Criswell, 2011) (although the higher end can extend up to 600 Hz for some facial muscles due to their smaller size and higher innervation ratio). Specifically, relevant to MEG/EEG recordings, O'Donnell et al. (1974) found that the peak frequency of the masseter muscle, involved in chewing, is around 50–60 Hz, whereas for frontalis, the muscle which controls wrinkling of the brow, it is 30–40 Hz. The lower band-limit of the activity of these muscles is around 15 Hz, while the high-end activity extends to well above 100 Hz (O'Donnell et al., 1974). Similarly, Goncharova et al. (2003) report frequencies around 20–30 Hz for frontal muscles and 40–80 Hz for temporal muscles. For posterior head muscles—sternocleidomastoids, splenius capitus, and trapezius—higher peak frequencies (~100 Hz) are reported, but these differ between muscles, direction and force of contraction as well as participants' sex (Kumar et al., 2003). The extraocular muscles that contain both striate and smooth fibres and control saccadic eye movements produce activity that peaks around 65 Hz (Yuval-Greenberg et al., 2008; Carl et al., 2012). Although peak frequencies of the various head muscles differ between muscles, the types of contraction and participants, one key feature to note is that in general the spectral bandwidth of muscle activity is broad.

The amplitude of muscle activity when recorded in temporal MEG/EEG electrodes and sensors can be ~1000 fT and 100 μV, respectively. This is several orders of magnitude larger than what one might expect from high-frequency activity, which can be less than 20 fT and 1 μV in MEG and EEG, respectively (Herrmann and Demiralp, 2005). The tiny size of these neural oscillations relative to the size of potential muscle artifacts is highly problematic. While large muscle artifacts can easily be screened and removed from data, small muscular artifacts can pass such screening and remain present in so-called artifact-free MEG/EEG recordings. The existence of muscular contamination of the EEG has been empirically shown in several elegant experiments that used neuromuscular blockade (Whitham et al., 2007, 2008). In this experimental model, EEG recordings were made during complete neuromuscular blockade with cisatracurium (which is functionally similar to curare). This allowed the EEG to be recorded with no EMG artifacts and to be compared to EEG recorded in a conventional way. These studies showed that even for electrodes near the center of the head, which are situated at a relative distance from the cranial muscles, the normal resting EEG shows significant contamination with EMG activity. The contamination of the frequency spectrum started around 20 Hz, such that at 40 Hz there was ~5 times more power in the non-paralyzed state, while at 80 Hz there was ~10 times more power. As one would expect for scalp-recorded muscle activity, the spatial topography of increased high-frequency power was maximal at the edges of the electrode montage. The levels of EMG contamination increased even further when participants were asked to perform cognitive tasks, as is common in cognitive neuroscience experiments (Whitham et al., 2008). While a previous study (Goncharova et al., 2003) had demonstrated the spectral and spatial topography of muscle artifacts using intentional contractions to contaminate the recordings, the neuromuscular blockade studies demonstrated that even “clean” resting EEG was heavily contaminated in the high-frequency range with broadband muscle activity. Unfortunately, an equivalent paralysis study has not been conducted with MEG; however, due to decreased volume conduction effects in MEG, one would predict significantly less resting artifact in central-parietal MEG sensors compared to EEG. This is due to the fact that the magnetic field falls off rapidly as distance increases to the primary dipole generator. Secondary volume currents are thought to contribute little to the external MEG field (see Hamalainen et al., 1993). This effect has recently been described in simultaneous MEG/EEG recordings following administration of the benzodiazepine secobarbitol (Claus et al., 2012) where EEG showed heavy contamination with muscle artifacts, particularly in frontal–temporal sensors, while the MEG was relatively clean, with some relatively localized peripheral contamination.

## The characteristics of high-frequency neural activity in MEG/EEG/iEEG

Having defined the basic characteristics of muscle artifacts in MEG/EEG, we now consider properties of neuronal high-frequency activity. The following summary is not a comprehensive review of all high-frequency MEG/EEG/iEEG papers; rather, it provides indicative examples that demonstrate the known diversity of high-frequency responses observed in the brain [other useful review articles the reader may wish to consult include (Fries, 2009; Jerbi et al., 2009b; Donner and Siegel, 2011)]. A particular emphasis is made on the studies that use intracranial EEG (iEEG), because in comparison to MEG/EEG studies, iEEG tends to be significantly less affected by artifacts, and can therefore indicate the morphology of responses that MEG/EEG studies might characterize. That said, because the transfer function between iEEG to MEG/EEG is not completely understood, it may be that some activity present in the iEEG is absent from the MEG/EEG.

### Types of high-frequency activity

It is useful to first define four types of high-frequency activity that are commonly described in the literature. Firstly, there is *resting* high-frequency activity, which is usually based on the frequency spectrum of the MEG/EEG measured while participants passively sit with their eyes open or closed. During the performance of tasks and/or presentation of stimuli with multiple trials, three further types of activity can be defined. *Induced* high-frequency activity is defined as increases or decreases in high-frequency amplitudes, which occur after, but are not phase-locked to, experimental events. This type of high-frequency activity is lost by as a result of time-domain signal averaging and may either occur briefly, or be sustained for extended periods of time. To recover this type of activity, the power on single trials must be computed prior to averaging across trials. Conversely, *evoked* high-frequency activity is computed by performing frequency analysis after calculation of the evoked response [with broader filters (>30 Hz) than usual for evoked-response studies]. To survive time-domain signal averaging, evoked high-frequency activity must be phase-locked to experimental events. In real data, high-frequency activity can exist on a continuum between evoked and induced, and metrics such as the phase-locking factor (Tallon-Baudry et al., 1997) can quantify the degree of phase-locking. Finally, there exist *steady-state* high-frequency responses, where responses in the high-frequency are elicited in a narrow-band by the temporal frequency characteristics of the stimuli presented.

### Visual cortex

The spectral properties of neural high-frequency activities in MEG/EEG recordings are the unknown phenomenon that experimenters seek to describe and manipulate. In the primary visual cortex, where high-frequency activity has been extensively described, the typical bandwidth tends to be narrower than the electromyogram. For example, in primary visual cortex, the full width at half maximum of the induced high-frequency response to grating stimuli is usually around 20 Hz, with peak frequencies generally ranging from 40 to 70 Hz (Hoogenboom et al., 2006; Muthukumaraswamy et al., 2010). However, the transient response (0–200 ms) that occurs with these stimuli can be of much higher bandwidth, often exceeding 50 Hz (Frund et al., 2007). Local field potential (LFP) recordings from cat V1 (Kayser et al., 2003, 2004) demonstrate that while grating stimuli create a narrow-band high-frequency response, the response to stimuli with richer spatial-frequency content (natural movies and pixel noise) is different. These data showed a lower frequency high-frequency response similar to the range seen in MEG/EEG studies. However, higher frequency oscillations extended from 100 to >200 Hz, with a relatively quiet area existing in the spectrum from around 80–100 Hz (Kayser et al., 2003). Thus, while high-frequency activity in the visual cortex is often reported as being relatively narrow-band, this is not necessarily the case for all stimuli.

### Motor cortex

In the primary motor cortex, movement-related high-frequency power increases have been found during a variety of motor tasks by a number of groups (Schoffelen et al., 2005; Ball et al., 2008; Cheyne et al., 2008; Donner et al., 2009; Muthukumaraswamy, 2010). For simple single-limb movements, these studies demonstrate that high frequencies in the MEG peak around 70–80 Hz, with a bandwidth of ~40 Hz, and that when recorded non-invasively, high frequencies generally do not extend beyond 100 Hz. Peak frequency and bandwidth vary across individuals and the limb moved (Cheyne et al., 2008). Interestingly, in iEEG recordings of primary motor regions, the bandwidth of movement-related increases in power extends up to ~180 Hz (Crone et al., 2006; Miller et al., 2007, 2009), and these power increases can be broadband (50–200 Hz). MEG/EEG techniques appear to be less sensitive to these higher frequencies elicited from the motor cortices.

### Auditory and somatosensory cortices

In auditory cortex, iEEG recordings from epilepsy patients demonstrate that broadband high-frequency responses are produced by primary auditory cortex (Edwards et al., 2005; Cervenka et al., 2013b). Recent comparison of iEEG (Griffiths et al., 2010) and MEG data (Sedley et al., 2012) demonstrate that MEG is able to capture these high-frequency induced oscillations with transient responses detectable from 40 to 150 Hz for simple pitch stimuli. For object-related sounds, induced responses in the 75–110 Hz band have been observed (Schepers et al., 2012). In the somatosensory cortex, nociceptive stimuli induce high-frequency activity between 60 and 95 Hz in both MEG (Gross et al., 2007) and EEG (Zhang et al., 2012). A short latency broadband response (10–40 ms, 50–200 Hz) is elicited by median nerve stimulation (Gaetz and Cheyne, 2003) that is very similar in time-frequency characteristics to the response reported in iEEG (Fukuda et al., 2008). Following tactile stimulation, induced high-frequency activity occurs in primary somatosensory cortex that is enhanced by cueing attention to the body part to be stimulated (Bauer et al., 2006).

### Steady-state high-frequency responses

**Figure 1:**
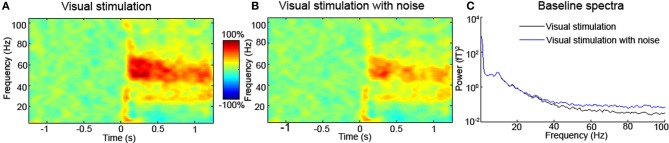
**(A)** Typical MEG source-level time-frequency response of a single participant to visual stimulation with a square-wave grating stimulus (data from Muthukumaraswamy et al., 2013). Equivalent EEG data look very similar (Muthukumaraswamy and Singh, 2013). In the time-frequency spectrum presented in **(B)**, white noise has been added to the channel prior to computation of the time-frequency response. The high-frequency response around 60 Hz is clearly attenuated in the presence of white noise, which similar to muscle activity has a broad bandwidth. Similar to this artificial addition of simulated noise, any experimental intervention (or use of different participant groups) that modulates baseline noise levels may appear to alter the induced high-frequency response. Units are percentage change from the pre-stimulus baseline for both (**A** and **B**). In **(C)** the baseline spectra (–1.2 to 0 s) are plotted for the original and original+ white noise channels. Inspection of these spectra reveals that high-frequency components are easily affected by noise. This broadband-added noise is similar to what might happen in the presence or absence of muscle artifacts. When differences in high-frequency are reported between interventions/participants/groups, comparison of the baseline spectra should be performed. Differences in the baseline may reflect artifactual or neural sources.

In several primary sensory areas it has been shown that high-frequency steady-state responses can be elicited by stimuli of high temporal frequency. This has been extensively described in the auditory system with the 40 Hz steady-state evoked response driven by 40 Hz tone pips (Pantev et al., 1991) and in the visual system with steady-state visual-evoked potentials (Regan, 1989; Herrmann, 2001). Similar effects have been described in the somatosensory system (Ross et al., 2012) at 40 and 60 Hz. From an analytical perspective, steady-state paradigms are relatively unproblematic as there is no evidence that these frequencies and their harmonics are represented in tight, band-limited, electromyographic frequencies. One popular way these techniques are used is in the investigation of high-frequency biomarkers of diseases, such as schizophrenia (Spencer et al., 2008; Spencer, 2011) and autism (Rojas et al., 2008, 2011). In such studies, it is still important that the baseline period is properly inspected for between-group differences, which can be caused by muscle artifacts that could confound the results (see Figure 1 and explanation). In epilepsy research, one stimulus-driven artifact is the photomyoclonic response caused by repeated contraction of frontal muscles in response to a repeating flash stimulus (~14–18 Hz) (Niedermeyer and Lopes Da Silva, 2005). The photomyoclonic response is maximal at frontal locations, and although stimulus-locked, readily identified as electromyogenic in nature.

### Pathological high-frequency oscillations in epilepsy

An important clinical application area is the non-invasive detection of the pathological high-frequency oscillations (40–200 Hz) that can accompany, but also be independent of (Andrade-Valenca et al., 2011), ictal and interictal spikes in various child and adult epilepsies (Kobayashi et al., 2004, 2010; Andrade-Valenca et al., 2011). While fast oscillations were originally thought to occur only in a small proportion of patients (<5%), several studies report detection rates of focal epilepsy that are significantly higher (Andrade-Valenca et al., 2011). These fast oscillations are potentially important because they appear to be generated near the zone of seizure onset (Jacobs et al., 2008; Zijlmans et al., 2009) and because the removal of the areas that generate pathological fast-oscillations have been shown to be a good predictor of surgical outcome (Jacobs et al., 2010; Wu et al., 2010). From a clinical perspective, initial, reliable, non-invasive detection of these oscillations is preferable to iEEG. Compared to other studies of high-frequency activity, investigations of the detection of pathological fast oscillations have the advantage that high-frequency oscillations can be recorded during (non-REM) sleep, which has significantly reduced levels of electromyographic contamination. While well-trained experts can distinguish the more spiky nature of artifactual EMG activity compared to the more sinusoidal fast oscillations, promising semi-automated algorithms are being developed for the detection of fast oscillations in scalp EEG (von Ellenrieder et al., 2012).

### High-frequency oscillations in association cortices

The association cortices, particularly the frontal and temporal association cortices, are some of the most problematic in which to examine high-frequency oscillations due to their proximity to artifact sources. iEEG studies have been very successful in describing high-frequency activity in association cortices, for example, broadband activity (50–150 Hz) in the frontal eye fields during pursuit eye-movements (Bastin et al., 2012). Similarly, in a study using frontal iEEG electrodes, 30–60 Hz high-frequency activity correlated positively with memory load in the Sternberg working memory task (Howard et al., 2003); and in another study, high-frequency activity during encoding predicted subsequent recall in frontal and more posterior electrodes (Sederberg et al., 2003). An MEG study recently found increased high-frequency activity, in a quite a narrow band (55–65 Hz), localized to SMA/preSMA during working long-term memory maintenance (Meeuwissen et al., 2011) [but *c.f.* with Brookes et al. (2011)]. High-frequency iEEG (50–200 Hz) activity occurs in frontal and temporal cortices in a number of language functions, including picture naming (Sinai et al., 2005), auditory word naming (Cervenka et al., 2013a), semantic processing (Crone et al., 2006), covert word repetition (Pei et al., 2011), and word production (Crone et al., 2001). In one very interesting iEEG study (Ossandon et al., 2011), broadband desynchronization of various nodes of the default mode network (including posterior cingulate cortex, temporal parietal junction, and medial prefrontal cortex) has been shown to occur during a visual-search task, suggesting that task-induced neural suppression of high-frequency activity can occur. In the future there will almost certainly be increasing attempts to characterize higher frequency MEG/EEG high-frequency responses in association cortices (up to 200 Hz), similar to those frequently seen in the iEEG literature.

In this brief summary, it has been demonstrated that the entire cerebral cortex appears to display a rich diversity of neuronal high-frequency responses. Methodologically speaking, efforts to identify high-frequency activity in lateral, frontal, and temporal cortices and also the cerebellum will be particularly problematic for MEG/EEG because these cortical areas lie close to the muscle areas in the head that were reviewed in section The Characteristics of Muscle Artifacts in MEG/EEG. Further, the broadband spectral responses that these iEEG studies describe are not dissimilar to the broadband spectral responses of muscles. Much like muscle artifacts, there is a large diversity of neuronal responses in terms of spatial, temporal, and spectral properties, as well as individual differences. For example, the frequency of the induced high-frequency oscillation (~40–70 Hz) varies with age (Gaetz et al., 2012), biochemistry (Muthukumaraswamy et al., 2009), anatomical features (Schwarzkopf et al., 2012), and even genetic factors (van Pelt et al., 2012). As such, there is no canonical high-frequency response or muscle response that can be easily applied as a discriminatory feature when deciding if the source of electromagnetic activity is neural or muscular.

## Saccade artifacts and high-frequency activity

One class of muscle activity that has received particular attention recently is the potential contamination of MEG/EEG high-frequency activity with miniature saccade artifacts (for example, microsaccades, saccadic intrusions). Traditionally microsaccades were defined as eye movements of amplitude less than 0.2° when attempting to maintain fixation; however, recently they have been re-defined as involuntary saccades that are produced while attempting to maintain fixation, with a one-degree upper limit on saccade size (Martinez-Conde et al., 2009). Regardless of type, when saccades occur, ocular muscles contract producing an electrical potential, called the pre-saccadic spike potential or, alternatively, the spike potential. For horizontal voluntary saccades, this manifests itself as an electrical potential occurring ~15–30 ms prior to saccade onset. Studies using dipole modeling suggest that the source of this activity lies in the rectus muscles of the eye (Thickbroom and Mastaglia, 1985). It is thought that the spike potential is generated by the synchronous recruitment of motor units creating a transient, summated, electrical potential in the extraocular muscles prior to saccade onset (Thickbroom and Mastaglia, 1987). In EEG, the spatial topography of the saccadic potential is a negative pole in frontal/ocular electrodes with posterior pole in occipitoparietal electrodes.

What has emerged is that a subset of so-called induced high-frequency responses in the EEG, which usually occur 200–400 ms after stimulus onset, are actually manifestations of the saccadic potential. Reva and Aftanas (2004) described a clear temporal co-incidence between occipital high-frequency activity in this time-period following presentation of picture stimuli and saccadic eye movements detected with electrooculograms (EOGs). Although an increase in parietal high-frequency activity was observed, no increase was seen in high-frequency activity in the bipolar EOGs; in other words, there was no increase in high-frequency power in these bipolar electrodes that can be used for saccade detection. This is because bipolar EOGs are optimal for detecting the corneo-retinal dipole generated by eyeball movement, where the cornea is positively charged relative to the retina (Keren et al., 2010). However, the saccadic spike potential is most prominent in peri-orbital electrodes and is of greatest amplitude when referenced to occipitoparietal electrodes (Thickbroom and Mastaglia, 1985). Trujillo et al. (2005) noted a similar effect when replicating a typical experiment of the time. In particular, they showed that parietal high-frequency activity (saccade artifacts) were reduced or eliminated by switching to a Laplacian referencing montage. This indicated that the high-frequency responses were due to contamination of the nose-tip reference selected for analysis rather than being due to an occipital cortex generator. Both of these studies used bipolar EOGs to detect saccades, which are relatively coarse in their ability to detect very small saccades (<1°). By combining high-resolution eye-tracking (~0.01° accuracy) with EEG, Yuval-Greenberg et al. (2008) demonstrated unequivocally that some of the induced high-frequency responses reported in the EEG are manifestations of saccadic artifacts. Later, the same researchers showed that the spike-potential artifact in EEG is present in most referencing schemes but is heavily attenuated by use of Laplacian montages (Keren et al., 2010). In this work, many of the detected saccades were of amplitude less than one degree, and while EOG electrodes can be used to detect a fair proportion of saccadic events (Keren et al., 2010), it does emphasize the importance of using eye-tracking techniques in EEG. Theoretically, posterior sensors of the MEG should be robust to spike-field contamination, and indeed it has been recently demonstrated that while the saccadic field does contaminate frontal and temporal sensors in MEG, parietal, central, and occipital sensors are free from this contamination artifact (Carl et al., 2012). Thus, saccadic spikes are still potentially problematic for MEG sources in frontal and temporal lobes. While MEG is more sensitive than EEG to high-frequency activity [at least for visual stimuli (Muthukumaraswamy and Singh, 2013)], it is a more problematic environment for monitoring fixation control. High-resolution eye-tracking in MEG is more technically challenging than in EEG because eye-tracking cameras must be kept somewhat distant to the MEG dewar to avoid equipment artifacts. Fortunately, this situation is changing with recent commercial improvements in eye-tracker technology, both in terms of camera speed and spatial resolution. These technical improvements will help with non-invasive investigations of the role high-frequency activity may play in eliciting microsaccades, or how high-frequency activity is affected by microsaccades (Bosman et al., 2009), which help to maintain the stability of images on the retina (Martinez-Conde et al., 2013). It is worth noting that the saccadic spike artifact can also occur in iEEG records, in which it is most prominent at electrodes in the temporal pole (Jerbi et al., 2009a). While the use of bipolar referencing and/or independent component analysis (ICA—see section Methods for Muscle Artifact Detection and Removal) largely attenuates the problem, some residual contamination exists for temporal electrodes (Kovach et al., 2011). For these reasons, use of eye-tracking in iEEG studies of these regions is advisable.

## Why use MEG/EEG to investigate high-frequency activity?

At this point the reader may be wondering whether attempting to use MEG/EEG to characterize high-frequency activity is a worthwhile exercise. Given the difficulties with artifact contamination in MEG/EEG and the improved sensitivity of iEEG described in section The Characteristics of High-Frequency Neural Activity in MEG/EEG/iEEG, it may seem that using MEG/EEG to non-invasively characterize high-frequency activity in humans is somewhat limited. It is clear that when characterizing high-frequency activity in humans (Lachaux et al., 2012), iEEG recordings have vastly superior signal-to-noise ratio compared to MEG/EEG. For EEG, the poorer signal-to-noise ratio is caused by attenuation and smearing of electrical potentials when they diffuse through the intervening (dura, skull, scalp) tissues to the surface recording electrode (Buzsaki et al., 2012); for MEG it is caused by the fact that the minimum distance between sources and the pick-up coils is greater than several centimeters (Hansen et al., 2010). Further, the potential spatial resolution of iEEG is vastly superior, as electrodes can potentially be tightly packed on the pial surface. While inter-electrode spacings of 3–10 mm are commonly used (Blakely et al., 2008; Jerbi et al., 2009b), it has been demonstrated that an interelectrode distances of ~1.25 mm would be ideal to avoid undersampling iEEG activity (Freeman et al., 2000). When the knowledge obtained from iEEG is combined with extracellular microelectrode recordings in non-human species, such as macaques (Ray et al., 2008), where LFP and multi-unit activity (MUA) can be obtained simultaneously from across cortical lamina (for examples Maier et al., 2010; Xing et al., 2012), highly detailed pictures of neuronal dynamics emerge. Non-invasive techniques seem to lose their competitive edge for detailed, mechanistic investigations of neuronal dynamics. However, invasive techniques have a number of shortcomings that are important to consider, and it is in this context that the usefulness of MEG/EEG become apparent.

Firstly, iEEG recordings are made only using patients with severe brain pathologies, usually uncontrolled epilepsies, and although efforts are made to avoid reporting experimental data from clearly epileptogenic cortex (Lachaux et al., 2012), iEEG electrodes by necessity are placed only surrounding the most likely symptomatic areas. Secondly, these patients have extensive medical histories, often beginning in childhood, which means that there is a large timeframe for cortical re-organization to occur. Thirdly, iEEG recordings usually only provide only a limited neurophysiological picture, because in that it is not possible to record simultaneously from the whole brain. Fourthly, because the spatial sampling of iEEG is idiosyncratic to each patient, it is difficult to conduct repeatability and/or group-level studies. Finally, patients undergoing iEEG procedures are usually medicated with drugs that have some interaction with high-frequency activity, which is thought to reflect the excitation-inhibition balance in the brain (Traub et al., 1996; Brunel and Wang, 2003; Bartos et al., 2007). While these drugs are usually withdrawn for iEEG procedures to facilitate seizure emergence, residual confounding effects on cortical excitability may remain. The obvious limitation of extracellular microelectrode recordings is that their use is largely restricted to non-human animals. In particular, key questions remain as to what extent abnormalities of high-frequency activity exist in neuropsychiatric disorders, such as, schizophrenia, autism, and depression. The degree of validity of animal models of these complex, human disorders is unclear.

The strength of MEG/EEG is not in attempting to compete with iEEG/microelectrode recordings in terms of characterizing microscopic neuronal dynamics, but in enabling the development of paradigms that allow neurophysiological function in humans to be probed non-invasively on a more global scale. Some key areas in which MEG/EEG can be used are; etiological studies of patient cohorts, large-scale genetic studies, development, ageing, and understanding the neuropharmacological basis of high-frequency activity (Bauer et al., 2012; Saxena et al., 2013). This can be done not only in an acute context but also to determine whether MEG/EEG can be used to predict or track successful treatment outcomes (for example Salvadore et al., 2010; Cornwell et al., 2012). A greater emphasis on paradigm design, quantification of repeatability, characterization of individual differences, and absence of artifacts in extracted neurophysiological parameters are critical if MEG/EEG are to be successfully used in these contexts.

## Methods for muscle artifact detection and removal

Having re-established the potential importance of MEG/EEG in measuring high-frequency activity, in this section we consider various promising methods for reducing and/or eliminating electromyographic artifacts from MEG/EEG data. One important observation already made is that electromyographic activity demonstrates considerable spectral variability in terms of amplitude, peak frequency, and bandwidth, depending on factors, such as the muscle(s) involved, contraction strength, lateralization, and the sex of the participant (Kumar et al., 2003). Moreover, it has been demonstrated that considerable individual variability exists in the amplitude, peak frequency, and bandwidth of high-frequency activity, at least in visual (Rols et al., 2001; Hoogenboom et al., 2006; Muthukumaraswamy et al., 2010) and motor cortices (Cheyne et al., 2008; Gaetz et al., 2010), where such variations have been extensively described. The net result of these physiological facts is that methods that attempt to use canonical, spatial, and spectral features to remove artifacts may be limited when it comes to eliminating EMG artifacts from MEG/EEG data (Shackman et al., 2009). The more promising approaches that have been used for attenuating muscle artifacts are based on spatial filtering, including the derivation of Laplacian montages (Fitzgibbon et al., 2013) for EEG, ICA (Shackman et al., 2009; McMenamin et al., 2011; Scheeringa et al., 2011), and beamformer source localization (Brookes et al., 2005; Litvak et al., 2011). The commonality of all these approaches is that sources, components, and channels are derived as weighted combinations of the recorded channel data. In each technique different assumptions are used to derive the weighting vectors.

In EEG research, calculation of the surface Laplacian, the second spatial derivative of the scalp-recorded EEG field (Nunez, 1981), uses weight vectors derived purely from the location of the electrodes on the scalp. Laplacians provide estimates of local current flux through the skull to the scalp and are therefore especially sensitive to sources local to the skull surface (Nunez and Srinivassan, 2006). Further, Laplacians perform best when cortical sources are from relatively small generators (Nunez and Srinivassan, 2006), as is likely to be the case for higher-frequency sources (Pfurtscheller and Copper, 1975). Using the neuromuscular blockade model, Fitzgibbon et al. (2013) demonstrated that Laplacian montages effectively reduce EMG contamination of centrally located EEG electrodes. While this seems promising, these results are difficult to reconcile with those of Goncharova et al. (2003), who found the Laplacian montages were ineffective at removing EMG artifacts. The difference between the results may lie in the exact details of the way EMG artifacts and their amplitudes were simulated. Nevertheless, the use of this method to eliminate artifacts from frontal and temporal sources remains problematic.

**Figure 2:**
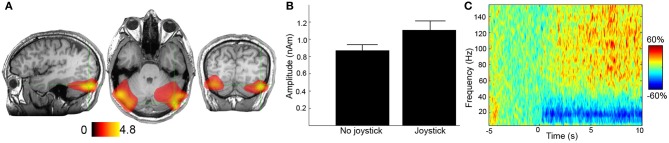
**Example of muscle artifacts in MEG data.** In this task (Kennedy et al., 2011), participants are asked to either track a moving object on screen with a joystick or simply observe the moving object. **(A)** The difference in 50–100 Hz source power is presented for the joystick and no-joystick conditions for a single participant. Units are pseudo *t*-values. **(B)** Peak-source amplitudes for the two conditions for the right hand source location. Based on panels **(A)** and **(B)** it would be tempting to speculate that tracking with the joystick has caused an increase in high-frequency activity in the bilateral cerebellar cortices; however, in panel **(C)** the reconstructed time-frequency spectrum is presented (10 s of tracking, baselined to 5 s of rest—units are percentage change from baseline). It is immediately apparent that there is broad bandwidth of the high-frequency activity in this virtual sensor. It is highly likely that this was caused by the increased postural activity of upper neck muscles, caused by the manipulation of the joystick. The lower-frequency beta-band desynchronization may represent a true difference in brain activity. This virtual sensor therefore contains a mixture of both brain and non-brain activity due to imperfect spatial filtering. Note: these data were recorded at 600 Hz with an anti-aliasing filter set at 150 Hz, the maximum frequency displayed here. Ideally, these data would have been sampled at a higher frequency to capture more bandwidth of the response. Recording of electromyograms from the neck muscles would also have been useful.

In ICA, the spatial filters are derived by producing the set of maximally temporally independent signals in the MEG/EEG data (Delorme et al., 2007). The components of the data can be inspected and those that resemble electromyogenic artifacts (broad bandwidth, peripheral distribution) can be projected out of the channel, leaving “clean” data. Disagreement exists in the literature about the effectiveness of ICA in removing EMG activity from data (Shackman et al., 2009; McMenamin et al., 2011; Olbrich et al., 2011). One downside of ICA use, is that it requires a process of artifact component selection. It is difficult to give operational definitions for artifact components (Gross et al., 2013) that can be universally applied. The decision regarding which components are artifacts that need to be removed from data is generally dependent on the neurophysiological expertise of the data analyst, which leads to problems with inter-observer reliability. Nevertheless, we can tentatively conclude that, (1) while partially effective, ICA-cleaned data may still contain residual EMG, and (2) analysis of the ICA time-courses may be preferable to analysis of ICA-cleaned data (Scheeringa et al., 2011). A technique most popularly used in MEG research, but also useable in EEG (Hipp et al., 2011), is beamformer-based source localization, where sets of spatial filters are created for each voxel in a predefined source space. The spatial filter at each voxel location is determined by minimizing the projected variance of a source at that location, subject to the linear constraint that the filter maintain a unity passband for a source at that location (Van Veen et al., 1997; Robinson and Vrba, 1999). While beamformer source-reconstruction images are not explicitly an artifact-removal algorithm, high-frequency artifacts in these images tend to localize to their source locations, making them relatively easy to spot in source-reconstruction images. Spatially filtered, source-space “virtual” sensors can then be subjected to subsequent analyses. However, for both ICA timecourses and beamformer virtual electrodes, caution must still be exercised because the channels are not necessarily artifact-free, particularly if the spatial filters are relatively coarse and the artifacts are relatively large (see Figure 2 for an example).

In summary, a number of techniques are available for the reduction of EMG artifacts, but at present, none of them are able to guarantee that the analysed data are free of high-frequency artifacts. Plenty of scope still exists for future methodological work to examine combinations and variations of these techniques as well as to attempt to automate artifact removal procedures. In particular, future work must ensure that artifact-removal techniques preserve the form of concurrent high-frequency activity. Given that well-described high-frequency responses can be obtained from primary cortices (auditory, visual, motor, somatosensory), new technical methods can and should be validated for a number of cortical locations at varying distances from artifact sources.

## Recommendations for the collection, analysis, and presentation of high-frequency MEG/EEG experiments

Given that muscle artifacts and high-frequency activity can share many spatial, temporal, and spectral properties, how can we avoid the mistakes of the past and reduce the number of artifactual reports that will appear in the literature? The most important point is that data are properly and fully presented; unfortunately, this has not always been the case. Below are some points to be considered:

1. Presentations of data that use statistical analysis only, without first presenting spectral or spatial representations should be avoided.
2. Presentation of (time-)frequency spectra is critically important. The spectrum should be presented in a way such that the full bandwidth of the high-frequency activity of interest is visible. For example, if high-frequency activity has a peak frequency of 50 Hz but its upper bandwidth extends above 80 Hz, then the spectrum should not be arbitrarily truncated in graphical representations at 80 Hz. The full bandwidth of high-frequency activity should always be represented. Broadband high-frequency activity may be a first indicator of electromyographic contamination.
3. Presentation of spatial maps (topographic maps and/or source localizations) for high-frequency activity is important. When broadband activity arises near the edge of the sensor/electrode montage, or the source solution space, this may be an indicator of electromyographic contamination (see Figure 2).
4. The techniques described in section Methods for Muscle Artifact Detection and Removal can be used to ameliorate muscle artifacts. In the case of EEG, it must be clear what reference has been used for the analysis. Re-referencing and particularly the use of Laplacian montages can be extremely informative in artifact identification and elimination.
5. When data are baselined to a pre-stimulus period, it is important that the baseline spectra are analysed as differences in the post-stimulus window can be driven by differences in the baseline period (see Figure 1 for details). If differences in the baseline high-frequency power spectrum are seen, they may be caused by differential electromyographic contamination in the comparison of interest. In this situation, presentation of the topography of the baseline power spectrum may be informative in determining whether baseline high-frequency power spectrum differences are of muscular origin.
6. In examining temporally sustained high-frequency activity, especially when the activity is induced, it is worth considering the extent to which the response appears to be “patchy” (in time-frequency representations). Patchy-looking responses may be indicative of muscle artifacts occurring in a subset of trials (see Figure 2).
7. Some consideration should be given to the amplitude of the signals (for example, on single trials) and to whether these signals are physiologically realistic for high-frequency brain activity.
8. It has recently been suggested that the inclusion of additional electromyographic electrodes over key muscle groups may be useful (Gross et al., 2013). This may be important in designing neurophysiological probes for high-frequency activity in frontal and temporal cortices. However, it may prove to be too complex due to the fact that the rich head musculature would require a large number of sets of bipolar electromyographic electrodes. In particular, one of the advantages of MEG in certain clinical groups is the quick and non-aversive application of the technique (for example, in autistic patients).
9. Collection of EOG is highly desirable for both MEG and EEG. Where feasible and appropriate, eye-tracking should also be considered (see section Saccade Artifacts and High-Frequency Activity). Again, this is particularly important for frontal and temporal sources.
10. At the time of acquisition, participants should be positioned comfortably in order to reduce postural muscle artifacts and, where appropriate, they should be instructed to relax their facial muscles. An investigation of the relative electromyographic contamination seen for supine vs. seated positioning in MEG/EEG would be useful.

As analytical techniques in MEG/EEG analyses—for example, neuronal mass modeling of data (Boly et al., 2012; Pinotsis et al., 2012), graph theory approaches (Stam, 2004; De Haan et al., 2012) and cross-frequency coupling (Canolty et al., 2006; Florin and Baillet, 2012; Voytek et al., 2013)—become progressively more complex, it is important that artifact-free data are being fed into these algorithms. For experiments involving responses that have been characterized many times, some of these requirements can be relaxed. However, for experiments that are designed to demonstrate high-frequency activity in new areas of the cortex or using novel paradigms, there is a greater obligation for a more complete presentation of the data. The scientific literature, even within the subfield of non-invasive neurophysiology, is vast and rapidly expanding. Unfortunately, the reality of the dissemination of scientific findings is such that once work is published and becomes embedded in the literature, there are few mechanisms that could prevent less experienced readers from citing publications that are known to report flawed results^2^. A reduction in the number of artifactual results that are reported is important to maintaining the long-term credibility of using MEG/EEG to study high-frequency activity.

### Conflict of interest statement

The author declares that the research was conducted in the absence of any commercial or financial relationships that could be construed as a potential conflict of interest.

## Footnotes

^1^Note: Various frequency-band definitions exist in the literature for gamma, low-gamma, high-gamma, high-frequency activity, as well as fast oscillations, etc. For the purposes of this review, high-frequency activity is defined as neural activity >~30 Hz, and as much as possible specific numerical frequencies are used rather than somewhat arbitrary frequency-band categorizations.

^2^Note: Recent years have seen the increased promotion and availability of cheap commercial products for online EEG control of, for example, computer games. Most of these systems are probably reactive to muscular rather than neural activity.
